# The Principles Involved in Focal Infection as Related to Systemic Disease*Read before the Section on Stomatology at the Sixty-Seventh Annual Session of the American Medical Association, Detroit, June, 1916. Printed in *Journal A. M. A.*, Sept. 16, 1916.

**Published:** 1916-10

**Authors:** Frank Billings

**Affiliations:** Chicago


### THE PRINCIPLES INVOLVED IN FOCAL INFECTION AS RELATED TO SYSTEMIC DISEASE.*

BY FRANK BILLINGS, M.D., CHICAGO.

We understand that an infectious disease is a condition due to the invasion of pathogenic micro-organisms, which are capable of multiplication within the tissues of the host.

The principles of infection, both local and general, may be discussed by a consideration of three chief factors:

1. The pathogenic micro-organisms and the conditions which modify their virulence and pathogenicity.

---

* Read before the Section on Stomatology at the Sixty-Seventh Annual Session of the American Medical Association, Detroit, June, 1916. Printed in *Journal A. M. A.*, Sept. 16, 1916.

2. The host or infected individual and the conditions which modify his susceptibility to infection.

3. The nature and result of the reactions between the infectious agents and the tissues of the host.

THE PATHOGENIC MICRO-ORGANISMS.

The pathogenic micro-organisms which may cause infectious diseases are certain fungi, unicellular animal life, the protozoa, and bacteria. We also recognize that certain infectious diseases are caused by a filterable virus of ultra-microscopic size. The subject of this paper will be limited to a discussion of infectious bacteria, with the exception that *endameba buccalis* will be considered in oral sepsis.

The objects of the paper will be quite as fully attained, too, by limiting the discussion to the usual pathogenic bacteria which cause focal infection, namely, the members of the streptococcus-pneumococcus group, staphylococci, gonococci, colon, diphtheria and tubercle bacilli, certain anaerobes and possibly other micro-organisms. Practically all of these may be found on the skin or mucous membranes, and if the surface is unbroken they may exist as harmless parasites. Apparently healthy individuals may be carriers of the bacilli of influenza and diphtheria and of streptococci, pneumococci and meningococci located on the mucous membrane of the tonsils, pharynx, nose and accessory sinuses. An individual who has had typhoid fever and occasionally one who has never had the disease may be a carrier of typhoid bacilli which may be discharged with the feces and urine. The bacteria named may bear an apparently harmless parasitic relation to the carrier, but if communicated to another individual may excite morbid reactions within the tissues of the new host.

The perpetuation of micro-organisms requires that they be transmitted from time to time to new hosts. That is accomplished by a direct route through the secretions and excretions of the mucous surfaces on which they live as parasites; through the pathologic products which they excite in infected tissues, and indirectly through the blood stream by way of the secretions and excretions. Here we are not concerned with those infectious diseases transmitted by blood-sucking insects. In direct transmission to new hosts the pathologic exudates must communicate with the outside surface, as in furunculosis, tonsillitis, sinusitis, gonorrhea, pyorrhea alveolaris, and bronchitis.

The transmission may be facilitated by direct individual contact, as in kissing and coitus; through approximate contact by inhaling air immediately charged with infected dust, droplets of saliva and sputum; by contract with infected articles, drinking and eating utensils, dental and surgical instruments, clothing, and many other forms of fomites; through infected food, especially milk, and by many other means. The tissues of the new host may be infected, therefore, through the mouth, air passages, skin or genito-urinary apparatus. The infection may be single, as, for example, in gonorrhea, or mixed, as may be seen frequently in diphtheria, with coincident streptococcus infection of the throat. A secondary infection is the invasion of pathogenic bacteria into the tissues previously infected. It is a type of mixed infection. Clinical experience and animal inoculation seem to show that bacterial association in mixed infection tends to increase the virulence of one or all of the species included in the microbic invasion. For example, appendicitis, which in most instances is caused primarily by the hematogenous invasion of bacteria, may become fulminating and rapidly gangreous through a secondary invasion of the injured tissues by the bacterial flora in the lumen of the appendix, usually colon bacilli and certain anaerobes.

A high degree of virulence of the infectious micro-organisms is liable to destroy the host, and thus directly diminishes the chances of the infectious agents to pass to new hosts. While the susceptibility of the host to a specific infection is an important factor in the result of the microbic invasion, still the laws which govern the perpetuation of pathogenic micro-organisms include adaptation to the conditions, which mean lessened pathogenicity and permit of parasitic existence. The permanency of the perpetuation of a given species will depend on the perfection of its adaption to the environment and the opportunity to pass to other hosts. As susceptible hosts diminish, through increased resistance, the infection becomes milder and mortality less. While the perpetuation of the existence of pathogenic micro-organisms is dependent on the attainment of a condition of parasitism and the opportunity to pass to the new hosts, there is proof that the infectious agents may regain pathogenic powers of a general and even of a special kind. The acquisition of a general or special pathogenicity with varying degrees of virulence may occur in various ways. Some of the species of bacteria under discussion are pathogenic for animals and may exist in the tissues of animals, as in man, as parasites. The bacteria existing as parasites in the animal may be pathogenic in the new host, man.

So, too, the mildly pathogenic or parasitic species may be communicated by man to a medium outside of the body, on which the bacteria may grow and attain pathogenic qualities and varying degrees of virulence for the host. Dairymen may infect milk with streptococci from their mouths and air passages. Milk is a good culture medium and in its streptococci and other bacteria may attain unusual virulence, and cause severe infection of the mucous membrane of the throat and digestive tract of susceptible persons who drink the unsterilized milk. The micro-organisms which have become parasitic in one human host may become pathogenic when communicated to a new host more susceptible to the species. The bacterial invaders, mildly pathogenic, may become virulent in a host suffering with general debility from any cause, or whose vitality is low or in whom the blood circulation is poor because of exposure to extreme cold or to severe fatigue. The conditions mentioned and other modifying influences may, among other things, affect the oxygen supply of the tissues of the host. The varying quantity of available oxygen in the tissues may be an important factor in determining the immediate or remote development of the micro-organisms in the tissues into harmless parasites or into virulent invaders of local or systemic tissues.

Schottmueller classified streptococci pathogenic for man, by growing them in solid blood-agar medium, into *S. hemolysans* (*longus*), *S. mucosus* and *S. viridans* (*mitior*). We have learned that any one of these types may change in general and special pathogenicity and the degree of virulence. *S. viridans,* so named because of the green-colored halo produced by the colonies on blood-agar, is usually of very mild pathogenicity for man. It is a very common surface parasite of the mucous membrane of the mouth and tonsil. Apparently this type of streptococcus may acquire special pathogenicity for the endocardium and especially for old valvular scars and cause enormous vegetations and thrombus formation on the heart valves. A person so affected suffers from a constant viridans streptococcemia. In other instances a green producing streptococcus of less virulence than the foregoing may be isolated from the infected tissues of the patients suffering from myositis, arthritis and other chronic diseases. Evidently the property of green color production on the blood-agar plates only affords a name, but does not signify the pathogenic character or virulence. The type may even lose the property of green color production on culture media.

By varying the character of the culture mediums, *S. hemolysans* may lose the property to hemolyze blood-agar medium. It may possess a hemolyzing property, and at the same time may vary in pathogenic characters. It may be pyogenic; may acquire a special pathogenicity and tropism for subcutaneous tissues, as in erysipelas without suppuration. The ability to hemolyze blood-agar does not necessarily indicate a special or general pathogenicity.

The variation of the members of the streptococcus-pneumococcus group in culture characteristics, biologic quality, in general and special pathogenicity and degree of virulence has been noted by many clinical bacteriologists. We know that these variations may be brought about in the laboratory, by serial animal inoculations and by modifications of the character of the media and the oxygen pressure in bacterial cultures.

Apparently like changes may occur in localized, confined infections, especially those due to the streptococcus-pneumococcus group. Here one may infer that the tissues involved in the focal infection act as a culture medium. The tissue mediums may be modified by variations in blood supply, consequently in oxygen tension, or unknown biochemic or other factors may alter the character of the confined infectious agents. Apparently in no other way may we explain the sudden onset of rheumatism after acute or during chronic tonsillitis; of streptococcus viridans endocarditis associated with alveolar abscess, and many other systemic infections, undoubtedly etiologically related to an existing chronic, localized and confined infection.

### THE HOST AND THE CONDITIONS WHICH MODIFY HIS SUSCEPTIBILITY TO INFECTION.

Man possesses a natural immunity to infection, but it is never absolute. The facility of infection of the person and the opportunity for the pathogenic micro-organisms to perpetuate themselves, depends in some degree on the characteristics of the infectious agents, as we have seen, but also on conditions peculiar to the host.

The age, environment, social condition, occupation, habits, domiciliary and occupational environment, climate, physical well-being are important factors in determining the susceptibility of the host to infection. It is not necessary to consider all of these factors, but a detailed discussion of some of them is important in the attempt to establish a clear idea of the principles involved.

The individual host must be considered first, because he becomes a carrier of infection the more readily in proportion to the lack of personal cleanliness, in the habitual overuse of alcohol and habit forming drugs, the practice of sexual immorality and other debility-producing habits. By contact with his fellow men in dwelling, workshop, and public conveyances and public congregations, he readily communicates the infectious parasites of which he is a carrier to others. The new hosts are more or less susceptible, dependent on the conditions which make for good or for bad defense, already mentioned. The facility of communication of the infection from host to host will be necessarily modified by the density of the population, by the hygenic conditions of the home, the workshop, the vehicles of transit, by the general conditions dependent on municipal control of the cleanliness of the streets, the water and the food supply and on the adequacy of the sewage disposal.

The person is also important as a host, in regard to the susceptibility to infection due to the modifying influence of age, occupation and the existing physical and mental conditions. The child is comparatively more susceptible to the contagious diseases. The lymphoid tissues of the child are relatively excessive, especially in the structures of the nose and pharynx. Therefore children suffer frequently with tonsillitis, coryza, sinusitis, otitis media and mastoiditis, both acute and chronic. The regional cervical lymph nodes may be secondarily infected from the foci named, and while the lymphadenitis may prevent immediate general infection, the infected glands may serve, sooner or later, as additional sources of systemic infection. The debility of old age, with the associated poor blood circulation, relatively low tissue nutrition and faulty metabolism, increases the susceptibility to infection and facilitates parasitism. Diseases due to faulty metabolism, like diabetes mellitus and gout, invite and prolong infection.

Exhausting chronic infectious diseases, like pulmonary tuberculosis, are frequently associated with secondary infection of pyogenic bacteria. The mixed infection promotes the virulence of one or all of the invading pathogenic micro-organisms with consequent harm to the host. Occupations which entail exposure to excessive fatigue, extremes of heat and cold and to air contaminated with mechanical and chemical irritants are important factors in the production of local and general infection.

### THE NATURE AND RESULT OF THE REACTIONS BETWEEN THE INFECTIOUS MICRO-ORGANISMS AND THE TISSUES OF THE HOST.

The entrance of pathogenic micro-organisms into the body of a susceptible host is characterized by local or general reaction between the invading parasites and the tissues of the host. Dependent on the virulence and genus of the invaders, the reaction in the tissues may result in exudates which may be serous, fibrinous, purulent diphtheritic, hemorrhagic, necrotic or proliferative. Local fibrinous and purulent exudates are apparent efforts of the tissues of the host to concentrate protective substances and physical barriers about the invading parasites to prevent extension of further infection. The walled-off infection may remain localized, the infectious parasites, adjusting themselves to a parasitic existence or local infection, may disappear under the influences of the defensive and offensive powers of the tissues, aided by medicinal or surgical management. On the other hand, the local infection may be coincident with general systemic infection or general infection may occur at some indefinite time subsequent to the local tissue invasion.

Systemic invasion from a local infection may be by way of the blood or lymph stream. The hematogenous route is undoubtedly the usual one. Practically always the regional lymph nodes are secondarily invaded from the primary local tissue infection. The lymph node infection may be subsequently a source of systemic infection by the hematogeneous route. On the other hand the secondarily infected lymph nodes may be the place of destruction of the invaders.

The reactions of the systemic tissues hematogeously infected are essentially the same as those mentioned under the local reactions of the tissues; that is, the production of serous, fibrinous, purulent, hemorrhagic or other inflammatory conditions. As already pointed out, these reactions are in the nature of defensive or offensive measures on the part of the tissues of the host to destroy the invading bacteria. The infectious micro-organisms also promote the formation within the tissues of the host of defensive and offensive substances, or antibodies. These substances, in the form of ferments, are apparently mobilized by the reaction between the invading bacteria and the tissues of the host. Immunologists are not in agreement as to the exact factors which enter into the reaction. All are in agreement, that all pathogenic bacteria do not cause the mobilization of a uniform number or of like kind of antibodies. The number and kind of antibodies found in the host in some infectious diseases, like typhoid fever, may overcome the invaders and afford a lifelong immunity to future infection. In other infectious diseases the defensive and offensive forces may overcome the present invasion, but afford no protection, or apparently may increase the susceptibility to future attacks, as in pneumonia and rheumatism.

The result of hematogenous invasion with pathogenic bacteria will be modified by the specific type, the virulence of the bacteria and also by the character of the anatomic structure and specific function of the tissue involved.

The type and virulence will decide the character of the tissue reaction as to the formation of a serous, fibrinous, purulent or other form of exudate. The more virulent the invaders the severer the reaction. The less virulent type will cause serofibrinous exudates. The tissues of the host are disturbed by the reactions, destructive in character

(abscess, necrosis, hemorrhage and gangrene) by virulent micro-organisms, and deprived of nutrition and oxygen, more or less, by the proliferation of the endothelium of the arterioles and the serous and fibrinous exudates by the less virulent invaders. The infection of a joint with virulent streptococci or virulent gonococci will cause purulent destructive arthritis. The infection with less virulent streptococci and gonococci will result, usually, in a chronic serous or fibrinous arthritis, with subsequent metabolic degenerative changes due apparently to the poor local nutrition, in consequence of the injury to the blood vessels and tissues of the joint. Very virulent bacteria are apt to cause local tissue destruction or to kill the host. That is, virulent bacteria may be ultimately destroyed by the severe reactions they excite in the tissues of the host.

On the other hand, pathogenic micro-organisms of low virulence, which gain access through the blood stream, excite mild reactions, which, however, lessen the supply of blood and oxygen to the tissues involved. This may create a condition favorable to the perpetuation of the invading bacteria in the tissues of the host. In this tissue medium the bacteria may continue to be pathogenic for an indefinite time or may become purely parasitic without apparent harm to the host. Chronic confined infection or even chronic general infection finds an explanation in the reactions excited in the tissues of the host by pathogenic bacteria of relatively low virulence. The natural anatomic structures of the teeth, dental alveoli, faucial tonsils, accessory nasal sinuses, seminal vesicles, prostate gland, fallopian tubes, gallbladder and other structures favor confined infection. When infected, the resulting morbid anatomic changes in all the tissues named add to the physical conditions which promote focal infection. This is well illustrated in chronic alveolar abscess. The invasion of the dental alveoli with *endameba buccalis* streptococci and other micro-organisms results in destruction of the peridental membrane and other tissues. The roots of the teeth deprived of periosteum become more or less devitalized and, with other local dead tissues, promote continued and aggravated confined infection. The structure of the faucial tonsils invites and promotes continued infection. An infected accessory nasal sinus does not as readily discharge the pathologic products of inflammation as the normal mucus secretion. The seminal vesicles infected with gonococci may retain them for years of time.

The function of infected specialized tissue will be usually disturbed in ratio to the degree of anatomic injury due to the invading bacteria. Hematogenous infection of the kidney may result in a general glomerulonephritis of a degree of severity entirely to suppress its function; a life infection of less severity may diminish the function, as manifested by the excretion of a scant, bloody albuminous and cast-containing urine. Periodic hematogenous infection of specialized tissues with bacteria of relatively low resistance results in repeated anatomic injury. The repeated injury to the blood vessels and other tissues may be so great finally as to result in malnutrition, and metabolic changes degenerative in character take place. Probably this is the sequence of events in chronic infectious nephritis, arthritis, myositis, pancreatitis and chronic infections of other organs.

The space allotted to this paper will not permit a discussion of the interesting tissue reactions, morbid anatomic changes and altered function which occur in infection of the stomach, subcutaneous tissues, the nerve ganglia, iris, thyroid gland and other specialized tissues.

### CONCLUSIONS.

The conclusions which may be drawn from this discussion of the principles involved in local and general infection are evident. The laws which govern the perpetuation of pathogenic micro-organisms involve a life of parasitism harmless to the host or of varying degrees of pathogenicity. Apparently any specific type of the bacteria discussed may attain the biochemic qualities which permit them to exist in the host as harmless parasites or as injurious agents possessed of a special or a general pathogenicity of varying virulence. The varying pathogenic qualities, special and general, may be acquired apparently in the host or in the passage from host to host (man or animals), or may be brought about in culture mediums.

Confined infection (focal) seems to be a site in which the infectious agents may attain specific pathogenicity chiefly in the nature of tissue tropism (elective tissue affinity). This special quality is not recognized necessarily by cultural characteristics. The power to hemolyze or to produce green color in blood-agar plates by some members of the streptococcus group does not necessarily indicate specific pathogenicity or degree of virulence. The special or general pathogenicity of the infectious agents of the focal infection, and the susceptibility of the host, measured by many factors cited in the paper, may determine the severity, acute or chronic, the extent, local or general, and the site, election of tissue, of the systemic infection. It is believed that these conclusions have been sustained by clinical observations and bacteriologic research.

